# Association between the urine tobacco metabolite levels and physical health in Chinese primary school students with secondhand smoke exposure

**DOI:** 10.3389/fpubh.2025.1600196

**Published:** 2025-07-17

**Authors:** Menghan Cheng, Miao Zhang, Wen Fang, Hong Qin, Yue Pan, Shengyi Chen, Guiqi Cao, Yao Zhang, Yu Lu

**Affiliations:** Center for Environment and Health in Water Source Area of South-to-North Water Diversion, School of Public Health, Hubei University of Medicine, Shiyan, Hubei, China

**Keywords:** second-hand smoke, urinary tobacco metabolites, physical health, primary school student, internal exposure

## Abstract

**Background:**

There is limited epidemiological evidence on the association between secondhand smoke exposure and growth and developmental problems in children and adolescents. This study aimed to explore the relationship between urinary levels of nicotine, cotinine, and trans-3′-hydroxycotinine and physical health outcomes in Chinese children.

**Methods:**

This cross-sectional study included 491 children aged 8 to 12 years from a primary school in 2023. Socio-demographic characteristics and lifestyle factors, including household routines and caregiver smoking status, were collected through a structured questionnaire. This information was subsequently analyzed to investigate correlations between urinary tobacco metabolite concentrations and physical health outcomes in children. Generalized linear regression models were applied to evaluate the relationships between urinary levels of nicotine, cotinine, and trans-3′-hydroxycotinine and key physical health indicators.

**Results:**

Among the 491 children, 168 were classified as overweight or obese, 134 had hypertension, 17 showed abnormal spirometry results, and 394 had low vision. The median urinary concentrations of nicotine, cotinine, and trans-3′-hydroxycotinine were 4.70 ng/mL, 3.91 ng/mL, and 5.33 ng/mL, respectively. After adjusting for potential confounders, urinary nicotine levels were positively associated with weight, body mass index (BMI), systolic blood pressure (SBP), and waist circumference. No significant associations were observed between urinary cotinine or trans-3′-hydroxycotinine levels and physical health indicators.

**Conclusion:**

Urinary nicotine levels were positively associated with weight, BMI, systolic blood pressure, and waist circumference. In contrast, no significant associations were found between urinary cotinine or trans-3′-hydroxycotinine levels and physical health indicators. These findings suggest that further longitudinal cohort studies are warranted to evaluate the potential of urinary cotinine and trans-3′-hydroxycotinine as reliable biomarkers for assessing and monitoring the physical health status of primary school children.

## 1 Introduction

The World Health Organization (WHO) reported that China has 316 million smokers, comprising almost one-third of the global smoking population. Smoking is widely acknowledged as a significant risk factor for numerous diseases, notably cardiovascular and pulmonary conditions as well as cancer. Furthermore, the WHO estimates that over 700 million non-smokers in China are subjected to secondhand smoke exposure, with 180 million of these individuals being children (1–3). Secondhand smoke, alternatively referred to as passive or environmental tobacco smoke, is the aerosol-filled air that results from the combustion of tobacco products such as cigarettes, pipes, hookahs, and e-cigarettes (4, 5). Cigarette smoke comprises over 5,000 chemicals, approximately 70 of which are identified as carcinogens. Nicotine and its primary metabolites, cotinine and trans-3′-hydroxycotinine, are recognized as reliable biomarkers for secondhand smoke exposure (6). Studies have shown that long-term exposure to secondhand smoke is a significant risk factor for various respiratory diseases in children, and its inhalation can lead to lung cancer, hypertension, and obesity in non-smokers (7–9). Primary school students’ physiological functions are not yet mature, making them highly susceptible to the effects of diet, mental health, and environmental conditions such as air quality, water quality and secondhand smoke (10). The eighth National Students’ Physical Health Research Report shows that the long-term trend in the growth and development of primary school students in China is generally developing favorably, and the height, weight, and other morphological development indicators of primary school students continue to grow, but there is also a decline in physical fitness. The detection rate of overweight and obesity among primary school students has been rising rapidly, and the rate of abdominal obesity has also risen sharply (11, 12). Hypertension is a major cause of many major human diseases and a risk factor for cardiovascular diseases such as coronary heart disease, congestive heart failure and stroke. In recent years, hypertension has shown a trend toward younger age; the prevalence of hypertension in children and adolescents has increased, and childhood hypertension is an independent risk factor for adult hypertension. In addition, the onset of early hypertension is associated with changes in cardiovascular target organs that predict cardiovascular consequences in adulthood and lead to substantial morbidity and mortality (13). In recent years, the detection rates of lung function abnormalities and poor vision have been on the rise (14, 15). Homes, schools, and public places are important places where primary school students are exposed to secondhand smoke (16). However, comprehensive and detailed research examining the precise correlation between secondhand smoke exposure and physical health among primary school students is notably scarce. In this study, we evaluated the association between secondhand smoke exposure and physical health, such as blood pressure, height, or weight levels, in non-smoking Chinese children. The status of secondhand smoke exposure was assessed subjectively by self-report questionnaires as well as objectively by urine nicotine, urine cotinine, and urine trans-3′-hydroxycotinine concentrations, quantifying the amount of passive exposure to tobacco smoke.

## 2 Materials and methods

### 2.1 Study population

A cross-sectional study was conducted in 2023 on children from a primary school in Shiyan City, Hubei Province, China. A total of 534 children aged 8–12 years old were included in the study to investigate the simultaneous impacts of secondhand smoke exposure on the growth and development indexes of School-age children. After excluding those with unrecovered questionnaires (*n* = 15, a recovery rate of 97.19%), insufficient physical examination (*n* = 14), and missing urine samples (*n* = 12), a total of 491 participants were eligible for further analysis. Demographic information such as age, sex, disease history, grade, caregiver smoking status, physical activity time, and dietary habits was collected by face-to-face interviews and questionnaires.

### 2.2 Physical examinations

#### 2.2.1 Height and weight measurements

Height measurements were conducted according to the technical specifications for student health examinations. Weight was measured in fasting subjects wearing light clothing, without shoes, and with an empty bladder to calculate BMI (kg/m^2^), using the same scale (Suhong, China) for all. Overweight and obesity among children were determined via sex and age-specific BMI, according to the national screening standard for overweight and obesity among school-age children and adolescents (17).

#### 2.2.2 Waist circumference measurement

When measuring the waist circumference, keep the body upright lower the arms naturally, relax the abdomen and breathe steadily circle the arms naturally, relax the abdomen and breathe steadily, circle the tape on a horizontal plane 1 cm above the navel, and pay attention to the tape close to the skin, repeat the measurement for three times, and take the average value when the reading is accurate to 0.1 cm.

#### 2.2.3 Blood pressure measurements

Participants were asked to rest for at least 5 min in a quiet room with moderate lighting before blood pressure measurements, then place their bare upper arms at the level of the heart. Their SBP and diastolic blood pressure (DBP) (measured to the nearest 1 mmHg) were measured using an electronic time sphygmomanometer (680CR-680B, Yuyue, China) when the individuals were seated and quiet; two measurements were taken at intervals of 30s between each measurement, and the results were averaged. Hypertension was diagnosed on systolic and/or diastolic pressure > = 95th percentile for sex, age, and height according to the reference value of the Chinese Child Blood Pressure References.

#### 2.2.4 Vital capacity measurement

The CHEST HI-101 Portable Spirometer, with both high validity and reproducibility, was used to measure vital capacity.

#### 2.2.5 Visual acuity measurements

During the vision examination, the students stood 5 meters from the lightbox, and the visual acuity of both eyes (first right, then left) was tested. Visual acuity measurements are specified in the Appropriate Technology Guidelines for Prevention and Control of Myopia in Primary School Children and Adolescents.

### 2.3 Urinary tobacco metabolite measurements

Spot morning urine samples were collected from each child in a 50-mL sterile polyethene bottle. All the collected samples were sent to the laboratory within 4 h and then stored at −80°C before further analysis. Before analysis, frozen urine samples were thawed at room temperature before centrifugation. A centrifuged urine sample is first combined with a 5ug/mL mixed internal standard application solution. This mixture is then diluted to 1 mL using water and thoroughly mixed. The sample is subsequently centrifuged at 12,000 rpm for 20 min before being filtered through a 0.22 μm nylon 66 pore membrane. The resulting filtrate is transferred to a brown vial, which is then inserted into a high-performance liquid chromatography-mass spectrometry (HPLC-MS/MS) instrument (Thermo Fisher, United States) to determine the concentration of the urine. Chromatographic conditions: Acquity UPLC BEH HILIC column (2.1 mm × 100 mm, 1.7 μm); column temperature: 40°C; flow rate: 0.2 mL/min; injection volume: 2 μL; mobile phase: 0.1% ammonia (A)-acetonitrile (B). Gradient elution conditions: 0 ~ 2 min: 10 ~ 60% B, 2 ~ 4 min: 60 ~ 60% B, 4 ~ 5 min: 60 ~ 100% B, 5 ~ 7 min: 100 ~ 100% B, 7 ~ 7 min: 100 ~ 10% B, 7 ~ 10 min: 10 ~ 10% B. Mass spectrometry conditions: Ionization mode: ESI positive ion mode; Ion transfer tube temperature: 325°C; Nebulizer temperature: 350°C; Needle voltage: 3000 V. To investigate potential non-linear associations and to facilitate the interpretation of the model, we categorized nicotine, cotinine, and trans-3′-hydroxycotinine in urine into tertiles according to their distributions (T1: lowest third, T2: middle third, and T3: highest third). This tertile-based classification is a method commonly used in epidemiological studies to assess dose–response trends and to minimize the impact of outliers (18, 19).

### 2.4 Covariates

The model incorporates significant potential covariates to refine the analysis. These include gender (boy or girl), age (continuous, years), caregiver’s smoking status (yes or no), fried food intake (occasional or frequent), meat intake (occasional or frequent), physical activity time (≤30 min/day, >30 min/day), and screen time (≤2 h/day, >2 h/day).

### 2.5 Statistical analysis

Data were double-entered and validated using EpiData version 3.1. Statistical analyses were performed using IBM SPSS 26 and GraphPad Prism 9.5. For continuous variables, median, interquartile spacing (IQR), and standard deviation were used to describe the basic characteristics of the participants; for categorical variables, frequencies and proportions were used to describe the basic characteristics of the participants. Wilcoxon signed rank tests were used for continuous variables and chi-square tests for categorical variables. Generalized linear regression models were used, with height, weight, body mass index, blood pressure, waist circumference, vital capacity, and visual acuity as dependent variables and nicotine, cotinine, and trans-3′-hydroxycotinine as independent variables. The significance level for all statistical tests was set at *p* < 0.05.

## 3 Results

### 3.1 Characteristics of study participants

The descriptive characteristics of the 491 participants are summarized in Table 1. The study encompassed 279 boys (56.8%) and 212 girls (43.2%), with a median age of 10 years. Boys exhibited higher BMI, waist circumference, vital capacity, meat consumption, physical activity duration, and screen time than girls (*p*-values < 0.05 for all comparisons). No significant differences were observed between genders in terms of age, weight, systolic blood pressure (SBP), diastolic blood pressure (DBP), and visual acuity for both eyes (*p*-values > 0.05 for all comparisons). Of the 491 participants, 168 were classified as overweight or obese, 134 presented with hypertension, 17 displayed abnormal spirometry results, and 394 had diminished vision. The median concentrations for nicotine, cotinine, and trans-3′-hydroxycotinine were 4.70 ng/mL (IQR: 2.43, 7.11 ng/mL), 3.91 ng/mL (IQR: 2.15, 7.37 ng/mL), and 5.33 ng/mL (IQR: 2.34, 12.82 ng/mL), respectively.

**Table 1:** Characteristics of study participants.

Covariates	Options	Total	Boy	Girl	*Z*-value or *χ*^2^	*p*-value
Age (years)		10(10,11)	10(10,11)	10(10,11)	−1.057	0.291
Height (cm)		143.80(138.90,149.70)	143.20(138.40,148.30)	145.50(139.10,151.60)	−2.449	0.014
Weight (kg)		37.80(31.70,45.30)	38.10(31.40,45.30)	37.70(32.00,45.30)	−0.280	0.779
BMI (kg/m^2^)		17.78(16.12,21.16)	18.10(16.30,21.60)	17.50(15.80,20.10)	−0.392	0.017
Overweight and Obesity (%)	Yes	168(34.20)	111(39.80)	57(26.90)	8.904	0.003
	No	323(65.80)	168(60.20)	155(73.10)		
SBP (mmHg)		104(95,112)	104(96,113)	104(94,111)	−1.113	0.266
DBP (mmHg)		68(63,74)	68(63,74)	68(63,74)	−0.297	0.766
Hypertensive (%)	Yes	134(27.30)	72(25.80)	62(29.20)	0.718	0.397
	No	357(72.70)	207(74.20)	150(70.80)		
Waist circumference (cm)		63(58,70.20)	64.60(59.40,72.20)	60.70(56.20,68.40)	−4.846	<0.001
Vital capacity		2,108(1754,2,454)	2,208(1891,2,553)	2013(1,647,2324.30)	−4.324	<0.001
Abnormal spirometry (%)	Yes	17(3.50)	9(3.20)	8(3.80)	0.108	0.742
	No	474(96.50)	270(96.80)	204(96.20)		
Vision left		4.70(4.30,50)	4.70(4.30,5.00)	4.80(4.40,5.00)	−0.632	0.527
Vision right		4.70(4.30,4.90)	4.60(4.30,4.90)	4.70(4.30,4.90)	−0.654	0.513
Low vision (%)	Yes	394(80.20)	227(81.40)	167(78.80)	0.509	0.476
	No	97(19.80)	52(18.60)	45(21.20)		
Nicotine (ng/mL)		4.70(2.43,7.11)	4.73(2.63,7.31)	4.65(2.29,6.81)	−0.920	0.358
Cotinine (ng/mL)		3.91(2.15,7.37)	4.08(2.26,7.46)	3.59(1.96,7.28)	−1.654	0.098
Trans-3′-hydroxycotinine (ng/mL)		5.33(2.34,12.82)	5.44(2.48,13.04)	5.16(2.33,12.39)	−1.079	0.281
Caregiver’s smoking status (%)	Yes	161(32.80)	100(35.80)	61(28.80)	2.731	0.098
	No	330(67.20)	179(64.20)	151(71.20)		
Fried food intake (%)	Occasional	395(80.40)	218(78.10)	177(83.50)	2.196	0.138
	Frequent	96(19.60)	61(21.90)	35(16.50)		
Meat intake (%)	Occasional	331(67.70)	170(61.40)	161(75.90)	11.659	<0.001
	Frequent	158(32.30)	107(38.60)	51(24.10)		
Physical activity time (min·d^−1^)	≤30	417(85.30)	225(81.20)	192(90.60)	8.341	0.004
	>30	72(14.70)	52(18.80)	20(9.40)		
Screen time (h·d^−1^)	≤2	343(69.90)	183(65.60)	160(75.50)	5.585	0.018
	>2	148(30.10)	96(34.40)	52(24.50)		

### 3.2 Associations between urinary tobacco metabolite levels and physical health

In Table 2, urinary nicotine, cotinine, and trans-3′-hydroxycotinine levels were higher in children whose caregivers smoked than those whose caregivers did not smoke (all *p-*values< 0.05). Both urine nicotine and trans-3′-hydroxycotinine levels differed significantly by hypertensive (*p*-value < 0.05), and the presence of meat intake (*p*-value< 0.05). Both urine cotinine and trans-3′-hydroxycotinine levels differed significantly by fried food intake (all *p*-values < 0.05). Urinary nicotine levels were higher in overweight and obese students than in those who were not overweight and obese.

**Table 2:** Associations between urinary tobacco metabolite levels and physical health.

Covariates	Options	Nicotine (ng/mL)		Cotinine (ng/mL)		Trans-3’-Hydroxycotinine (ng/mL)	
		Median (IQR)	*P*-value	Median (IQR)	*P*-value	Median (IQR)	*P*-value
Sex	Boy	4.73(2.63,7.31)	0.358	4.08(2.26,7.46)	0.098	5.44(2.48,13.04)	0.281
	Girl	4.65(2.29,6.81)		3.59(1.96,7.28)		5.16(2.33,12.39)	
Overweight and Obesity	Yes	5.10(2.64,9.05)	0.050	4.05(2.31,7.34)	0.471	5.78(2.57,12.87)	0.108
	No	4.50(2.30,6.60)		3.80(2.10,7.40)		5.00(2.27,12.82)	
Hypertensive	Yes	5.20(3.17,9.21)	0.010	4.29(2.24,7.74)	0.081	6.34(2.63,13.37)	0.023
	No	4.51(2.15,6.82)		3.67(2.10,7.15)		5.00(2.24,12.54)	
Abnormal spirometry	Yes	4.64(3.00,10.57)	0.678	4.08(2.00,6.61)	0.794	4.94(2.61,8.61)	0.575
	No	4.72(2.39,7.09)		3.86(2.15,7.38)		5.34(2.33,12.90)	
Low vision	Yes	4.57(2.45,6.96)	0.447	3.93(2.16,7.46)	0.243	5.34(2.36,12.97)	0.390
	No	5.13(2.39,8.06)		3.76(2.13,6.21)		5.24(2.22,12.51)	
Caregiver’s smoking status	Yes	5.66(3.41,8.69)	<0.001	4.77(2.74,7.91)	<0.001	9.24(3.43,13.45)	<0.001
	No	4.35(2.06,6.54)		3.43(1.95,7.06)		4.43(2.11,12.44)	
Fried food intake	Occasional	4.91(2.73,7.30)	0.109	4.14(2.29,7.47)	0.001	6.06(2.51,13.02)	<0.001
	Frequent	3.66(2.13,6.71)		2.75(1.76,4.66)		3.41(1.79,10.71)	
Meat intake	Occasional	4.52(2.22,6.95)	0.037	4.02(2.19,7.44)	0.199	5.86(2.52,13.00)	0.020
	Frequent	5.19(2.93,7.66)		3.49(1.97,6.91)		4.11(2.02,12.32)	
Physical activity time (min·d^−1^)	≤30	4.70(2.38,7.45)	0.859	3.76(2.13,7.32)	0.386	5.00(2.33,12.71)	0.285
	>30	4.82(2.95,6.69)		4.33(2.37,7.54)		6.20(2.91,13.17)	
Screen time (h·d^−1^)	≤2	4.63(2.41,7.00)	0.340	3.67(1.94,7.34)	0.087	5.00(2.27,12.75)	0.406
	>2	4.98(2.46,7.88)		4.12(2.41,7.42)		5.61(2.59,12.96)	

### 3.3 Subgroup analyses of tobacco metabolite levels and physical health

**Figure 1:**
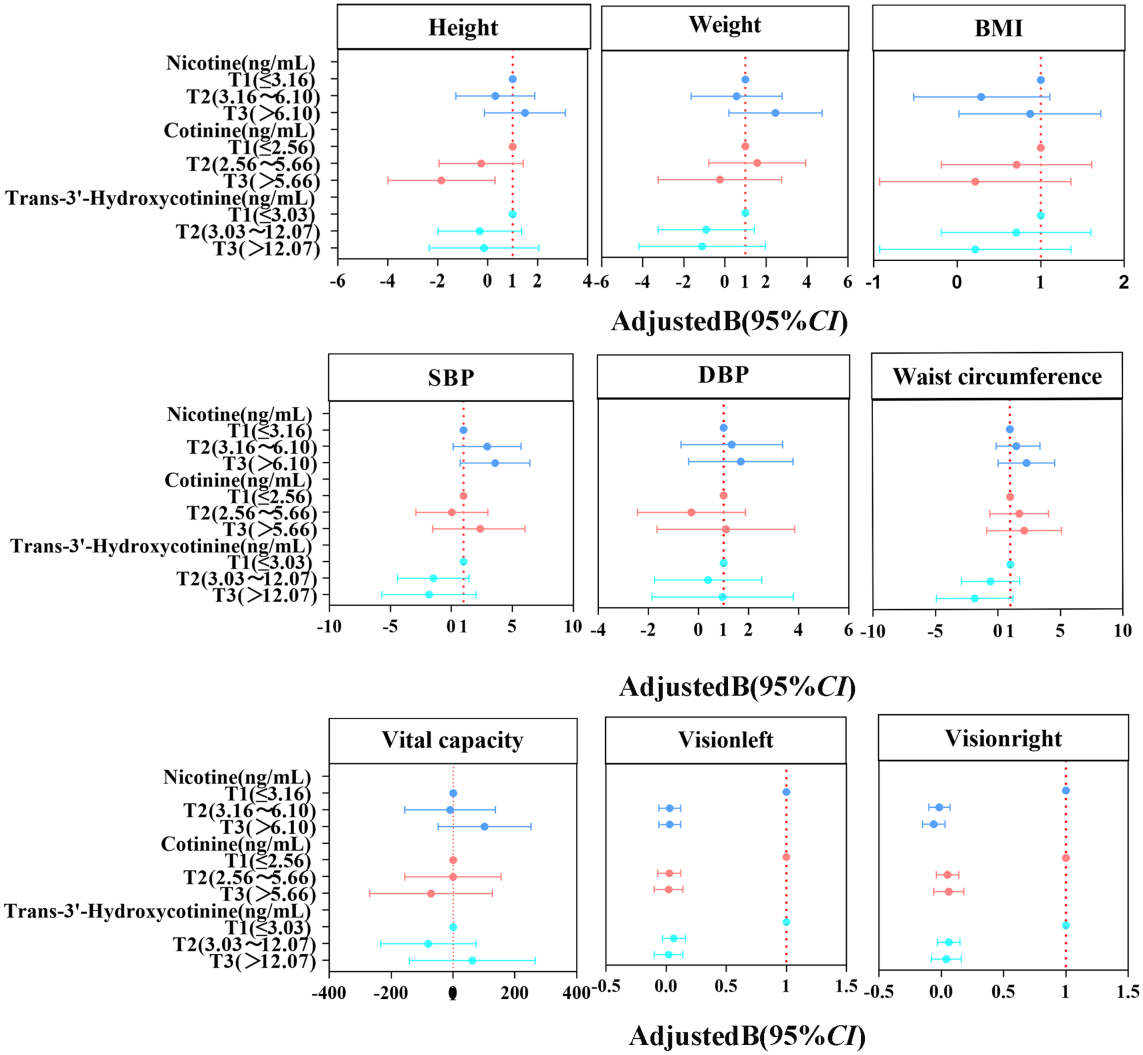
Generalized linear regression model forest plot. Adjusted for sex, age, caregiver’s smoking status, fried food intake, meat intake, physical activity time, screen time.

In Table 3, generalized linear regression was used to analyze the effects of tobacco metabolites on physical health in primary school students. The concentrations of nicotine, cotinine, and trans-3′-hydroxycotinine in urine were divided into three groups each, with the lowest concentration group, T1, serving as the reference. In the initial model, a positive association was observed between urine nicotine and several variables in primary school students, namely height, weight, BMI, SBP, DBP, waist circumference, and vital capacity (all *p*-values < 0.05). Conversely, urinary cotinine and trans-3′-hydroxycotinine showed a negative association with height in the same demographic (all *p*-values < 0.05). The model was adjusted to account for various socio-demographic and lifestyle factors, including sex, age, caregiver’s smoking status, consumption of fried and meat foods, physical activity duration, and screen time. Urinary nicotine was borderline significantly associated with height (*p* = 0.049). Urinary nicotine was positively associated with weight, BMI, SBP, and waist circumference (all *p-*values < 0.05). Urinary cotinine and trans-3′-hydroxycotinine were not significantly associated with physical health (all *p-*values > 0.05). Both in the original and corrected models, Urine nicotine, cotinine and trans-3′-hydroxycotinine were not significantly associated with visual acuity in primary school students (all *p-*values > 0.05). As shown in Figure 1, the distribution of urinary tobacco metabolite levels in relation to the physical health of primary school students after adjusting for confounders was shown by forest plotting.

**Table 3:** Subgroup analyses of tobacco metabolite levels and physical health.

Physical health	*β* (95%*CI*)												
	Nicotine (ng/mL)					Cotinine (ng/mL)				Trans-3’-hydroxycotinine (ng/mL)			
	Model	T1(≤3.16)	T2(3.16 ~ 6.10)	T3(>6.10)	*P* for tends	T1(≤2.56)	T2(2.56 ~ 5.66)	T3(>5.66)	*P* for tends	T1(≤3.03)	T2(3.03 ~ 12.07)	T3(>12.07)	*P* for tends
Height	Model1	1	2.16(0.43,3.89)	4.33(2.63,6.04)	<0.001	1	−0.54(−2.43,1.35)	−3.85(−6.23,-1.48)	<0.001	1	−1.34(−3.20,0.53)	−3.27(−5.66,-0.88)	0.01
	Model2	1	0.30(−1.28,1.88)	1.49(−0.14,3.11)	0.049	1	−0.27(−1.95,1.42)	−1.86(−4.00,0.29)	0.060	1	−0.32(−1.99,1.35)	−0.16(−2.35,2.04)	0.943
Weight	Model1	1	2.32(0.07,4.57)	5.22(3.00,7.44)	<0.001	1	1.64(−0.83,4.10)	−1.73(−4.83,1.36)	0.138	1	−1.69(−4.12,0.74)	−3.33(−6.44,-0.21)	0.058
	Model2	1	0.57(−1.65,2.78)	2.46(0.19,4.74)	0.014	1	1.58(−0.78,3.93)	−0.24(−3.25,2.76)	0.629	1	−0.91(−3.25,1.43)	−1.11(−4.19,1.97)	0.572
BMI	Model1	1	0.61(−0.22,1.44)	1.44(0.62,2.26)	<0.001	1	0.81(−0.11,1.72)	0.002(−1.14,1.15)	0.716	1	−0.42(−1.32,0.48)	−0.73(−1.88,0.43)	0.287
	Model2	1	0.27(−0.52,1.11)	0.88(0.02,1.75)	0.022	1	0.71(−0.19,1.61)	0.22(−0.93,1.36)	0.932	1	0.71(−0.19,1.6)	0.22(−0.93,1.36)	0.518
SBP	Model1	1	3.50(0.79,6.21)	4.52(1.85,7.20)	0.003	1	−0.01(−2.97,2.96)	1.35(−2.37,5.08)	0.405	1	−1.90(−4.83,1.02)	−2.75(−6.50,0.10)	0.259
	Model2	1	2.94(0.16,5.73)	3.59(0.73,6.45)	0.033	1	0.04(−2.92,3.00)	2.6(−1.52,6.04)	0.186	1	−1.48(−4.42,1.46)	−1.831(−5.70,2.03)	0.517
DBP	Model1	1	1.55(−0.43,3.52)	2.28(0.33,4.23)	0.032	1	−0.25(−2.41,1.92)	0.50(−2.22,3.21)	0.599	1	0.32(−1.82,2.45)	0.60(−2.14,3.33)	0.634
	Model2	1	1.32(−0.70,3.35)	1.69(−0.40,3.77)	0.169	1	−0.28(−2.44,1.87)	1.09(−1.66,3.84)	0.329	1	0.38(−1.76,2.52)	0.96(−1.86,3.78)	0.466
Waist circumference	Model1	1	1.78(−0.43,3.99)	3.35(1.17,5.53)	0.001	1	2.02(−0.40,4.44)	1.67(−1.36,4.71)	0.364	1	−1.03(−3.41,1.36)	−2.33(−5.39,0.74)	0.171
	Model2	1	1.21(−0.10,3.41)	2.31(0.05,4.58)	0.034	1	1.73(−0.62,4.07)	2.12(−0.88,5.10)	0.191	1	−0.59(−2.91,1.74)	−1.86(−4.92,1.20)	0.250
Vital capacity	Model1	1	112.38(−39.42,264.18)	278.53(128.66,428.39)	<0.001	1	0.76(−165.43,166.94)	−162.23(−370.79,46.33)	0.063	1	−147.20(−311.03,16.62)	−79.31(−289.38,130.76)	0.772
	Model2	1	−9.48(−155.57,136.6)	101.91(−48.32,252.14)	0.140	1	−0.33(−155.86,155.20)	−71.27(−269.64,127.10)	0.322	1	−79.74(−233.99,74.51)	62.34(−140.66,265.33)	0.356
Vision left	Model1	1	0.05(−0.04,0.13)	0.05(−0.04,0.13)	0.232	1	0.02(−0.07,0.12)	0.004(−0.113,0.122)	0.832	1	0.05(−0.05,0.14)	−0.02(−0.14,0.10)	0.547
	Model2	1	0.03(−0.06,0.12)	0.03(−0.06,0.12)	0.497	1	0.03(−0.07,0.12)	0.02(−0.10,0.14)	0.621	1	0.06(−0.03,0.16)	0.02(−0.10,0.14)	0.993
Vision right	Model1	1	0.009(−0.076,0.095)	−0.02(−0.11,0.06)	0.702	1	0.05(−0.04,0.14)	0.05(−0.07,0.17)	0.394	1	0.04(−0.05,0.13)	−0.003(−0.12,0.12)	0.812
	Model2	1	−0.02(−0.10,0.07)	−0.06(−0.15,0.03)	0.240	1	0.05(−0.04,0.14)	0.06(−0.06,0.18)	0.270	1	0.06(−0.03,0.15)	0.04(−0.08,0.16)	0.657

Model 1: unadjusted.

Model 2: adjusted for sex, age, Caregiver’s smoking status, fried food intake, meat intake, physical activity time, screen time.

*P* for tends across three-quarters of tobacco metabolites were obtained by including the median of each three-quarter as a continuous variable in generalized linear regression mode.

## 4 Discussion

In this cross-sectional population-based study, we evaluated the relationship between urine nicotine, cotinine, and trans-3′-hydroxycotinine and physical health in a population of primary school students (height, weight, BMI, SBP, DBP, waist circumference, vital capacity and vision) in Shiyan City, Hubei Province, China. After adjusting for confounding variables, urine nicotine levels were positively associated with height, weight, BMI, SBP, and waist circumference; urinary cotinine and trans-3′-hydroxycotinine levels were not associated with physical health indicators. Similar to first-hand smoking, secondhand smoke is harmful to the lungs, heart, and blood vessels. Therefore, minimizing secondhand smoke exposure is a global health target, and the early detection and prevention of secondhand smoke exposure are essential.

In this study, we found that urine nicotine was positively associated with height, weight, BMI, SBP, and waist circumference in the adjusted model. Urinary nicotine was borderline significantly associated with height (*p* = 0.049), a finding that may be influenced by family economic status and environmental factors and does not directly reflect causality; therefore, further mechanistic studies should be conducted. Smoking status was regarded as the independent variable in earlier studies (20), and there are limited reports on the association between nicotine in urine and obesity. In a Western study on secondhand smoke, overweight and obesity elevation were associated with increased urine nicotine levels among those in contact with secondhand smoke (21). Based on data from the Southern California Child Health Study, secondhand smoke exposure was also found to be positively associated with BMI (22). Daily exposure to secondhand smoke is associated with obesity in adolescents, found the study, which was based on data from the Global Health and Wellness Survey of 88,209 adolescents aged 12–15 years in 38 low- and middle-income countries (23). These findings are consistent with the findings of the present study, suggesting that exposure to secondhand smoke is associated with an increase in BMI among children and adolescents. However, some scholars have arrived at different conclusions. A previous study in the United States found that adolescent weight and BMI are negatively associated with active smoking (24). The mechanism may be related to nicotine’s activation of melanocortin-4 receptors in hypothalamic POMC neurons for appetite reduction and weight control (20). Studies have found that this may be attributable to the heterogeneity of the population (25). However, some studies dispute this conclusion: firstly, the neural pathways that regulate hunger and satiety are intricate and exhibit varied responses to nicotine. Animal studies have found an increase in hypothalamic neuropeptide Y (a potent orexigenic neuropeptide) in mice (Balb/c mice) after 7 weeks of nicotine inhalation (2 cigarettes, 2 times/day, 6 days/week for 7 consecutive weeks), suggesting that nicotine may have a facilitating effect on increased appetite (26). Mother-infant cohort studies have shown that infants of mothers who smoked during pregnancy had relatively more body fat and less lean body mass, and that this association persisted after correcting for demographic characteristics, lifestyle, and infant birth weight (27, 28). A recent study found that urinary nicotine levels were positively correlated with SBP, consistent with the findings of this study (29, 30). Nicotine can raise blood pressure through various biological mechanisms such as its sympathomimetic action, modulation of the renin-angiotensin system, and upregulation of arginine vasopressin and endothelin-1 (31–33). In animal experiments, Dikalov et al. (34) found that systolic blood pressure was significantly elevated in mice after exposure to cigarette smoke compared to unexposed mice, and the prevalence of hypertension also rose. In addition, a study found that the prevalence of waist circumference was higher among secondhand smoke-exposed participants (35). A prospective Health Outcomes and Environmental Measurement Study found that postnatal secondhand smoke exposure was not only associated with increased BMI and waist circumference in children, but also significantly increased body fat, lean body mass, and visceral fat (36). This finding may result from metabolic disturbances induced by tobacco exposure. Nicotine affects central adiposity through mechanisms such as sympathetic activation, insulin resistance and cortisol regulation. In addition, secondhand smoke exposure is often associated with poor household lifestyle factors that may further contribute to the accumulation of abdominal fat. In the present study, there was no significant effect of urine nicotine levels on visual acuity in primary school children. In America, a study showed no statistically significant difference in vision left and vision right between those who were exposed to secondhand smoke (37). Given that primary school students are in a pivotal phase of visual development, their visual sensitivity is predominantly influenced by genetic factors, environmental conditions, and established eye habits. Consequently, the impact of nicotine on visual sensitivity might be overshadowed by these determinants. Notably, nicotine predominantly affects the cardiovascular and nervous systems, with its direct influence on the eyes being relatively limited.

Several mechanisms have been proposed, such as numerous compounds found in smoke (e.g., nicotine) have negative endocrine effects that could lead to insulin resistance and metabolic imbalance. Nicotine activates nicotinic acetylcholine receptors (nAChRs) on the membranes of neuronal cells, endothelial cells, epidermal cells, and tumor cells, which in turn leads to cell proliferation, angiogenesis, surface-mesenchymal metastasis, invasion, and promotion of non-small-cell lung cancer cell growth. Nicotine also stimulates SCF cytokines, which can lead to self-renewal and differentiation of a variety of stem cells, leading to tumorigenesis (38). Cigarette smoke may also produce some biological mediators of inflammation through its effect on immune-inflammatory cells, and in turn, inflammation may increase the risk for obesity (23). Excitation of the sympathetic nerve underlies the effect of nicotine on blood pressure. There are two main mechanisms by which nicotine boosts sympathetic activity. The first mechanism demonstrates that nicotine acts directly on the central nervous system to activate the sympathetic nerves. In the central nervous system, specifically in the medulla, the rostral ventrolateral medulla is responsible for the basal and reflex control of sympathetic activity (39). The second mechanism involves the indirect activation of sympathetic nerves through the inhibition of nitric oxide production. A lowered nitric oxide level suppresses the effect of central sympathetic outflow (40). In addition, Passive smoking exposure leads to elevated levels of tobacco metabolites in the body, and the cumulative effect of nicotine on body weight may lead to insulin resistance and accumulation of abdominal fat (41). The mechanism of nicotine toxicity is not completely delineated. A substantial body of evidence suggests the participation of oxidative stress, reactive oxygen species, lipid peroxidation, and DNA damage, as well as the protective role of antioxidants. During the past 14 years, the hypothesis has enjoyed substantial support. Increasing evidence points to a role for oxidative stress in toxicity by nicotine entailing major body organs, including the lung, cardiovascular system, central nervous system, liver, kidney, testes, ovary, pancreas, and esophagus (42).

In the unadjusted model, urinary nicotine levels were positively associated with vital capacity. This result may be due to confounding, without considering covariates. A cohort study proves that excessive nicotine intake is negatively associated with vital capacity (43). Another study found that nicotine metabolism increased the risk of COPD, lung cancer, and lung function (1). This may be since both the particulate and gaseous phases of tobacco smoke are tumor-inducing, and tobacco also contains pro-carcinogens and pro-cancer agents. The development of squamous and small cell undifferentiated carcinomas is closely related to smoking status, and the risk of lung cancer increases with the number of cigarettes smoked per day and the number of years of smoking. Therefore, to further identify the causes, such confounders must be controlled in conjunction with the cohort study to assess the effect of nicotine exposure on children’s vital capacity. Reducing children’s exposure to tobacco-related carcinogens is essential to ensuring their healthy development.

Nicotine, the main psychoactive compound in cigarettes, is primarily inactivated by the liver CYP2A6 enzyme cotinine, which undergoes further CYP2A6-mediated metabolism to trans-3′-hydroxycotinine (44, 45). By using nicotine, cotinine, and trans-3′-hydroxycotinine biomarkers, we were able to assess how tobacco smoke affects the physical fitness status of primary school students, which may partially explain the differences in the physical fitness status effects observed among nicotine, cotinine, and trans-3′-hydroxycotinine in primary school students. After adjusting for covariates such as gender and age of the primary school students, this study found no association between cotinine and trans-3′-hydroxycotinine on the physical health status of primary school students. Probably because cotinine and trans-3′-hydroxycotinine are products of nicotine metabolism, their mechanism of action in the body is different from that of nicotine, and they may not have a direct effect on health. However, a study suggests that cotinine and trans-3′-hydroxycotinine are present in the body for a longer period than nicotine, making it easier to reflect ongoing secondhand smoke exposure and especially useful in measuring exposure in children (46). In recent years, the prevalence of tobacco exposure detected by urinary cotinine has been higher than with nicotine use, possibly due to the ability of cotinine to provide more stable and long-term exposure information (47). Therefore, further studies are needed to assess whether urinary cotinine and urinary trans-3′-hydroxycotinine can be used as indicators to quantify and monitor the health effects of smoke exposure in children.

The main novelty of our study is that it is the first reported evaluation of the association between tobacco metabolite levels and physical health indicators using the objective method. We believe that compared with the second-hand smoke exposure of school-age children obtained by self-report questionnaire, the exposure level of nicotine, cotinine, and trans-3′-hydroxycotinine in urine can reduce the bias caused by the inauthenticity of information to a certain extent. The main limitation of this study is its cross-sectional nature, we are unable to draw conclusions regarding the temporality and causality of exposure and outcome. Secondly, as nicotine, cotinine, and trans-3′-hydroxycotinine have limited half-lives, we had some limitations in assessing the effects of timing and prolonged exposure to second-hand smoke on health status. Additionally, the basic information for this study was collected through a questionnaire, which may be subject to recall bias. Additionally, while some confounding factors were adjusted for in the correlation analysis, the potential influence of other unknown factors, such as genetic predispositions and family socioeconomic status, could not be entirely ruled out. Finally, the current study possesses certain limitations concerning the source and representativeness of the sample. Future research could augment the generalizability of these findings by broadening the sample to include children from diverse regions and backgrounds. Future studies with a longitudinal design would provide better insight into the impact of tobacco metabolite levels on major physical fitness indicators in children. It is important to assess the effect of the timing of exposure, whether there are critical time windows of susceptibility to tobacco smoke exposure, and whether these sensitive time windows are in utero, early childhood, late childhood, or adolescence.

## 5 Conclusion

We performed a cross-sectional, population-based study of healthy children who had never smoked and who were without known diseases to evaluate the relationship between tobacco metabolite levels and physical health. The rise in urine nicotine levels was positively correlated with height, weight, BMI, SBP, and waist circumference. Urinary cotinine and trans-3′-hydroxycotinine levels were not associated with physical health indicators. Thus, tobacco exposure is harmful to many body systems. Reducing exposure to secondhand smoke promotes the healthy development of primary school students by focusing on prevention.

## Data Availability

The data are not publicly available due to their containing information that could compromise the privacy of research participants. equests to access the datasets should be directed to Menghan Cheng, 2252060857@qq.com.
