# Supplementary material for: Association between the urine tobacco metabolite levels and physical health in Chinese primary school students with secondhand smoke exposure
Source: Front Public Health. 2025 Jul 17;13:1600196. doi: 10.3389/fpubh.2025.1600196 (PMC12312655; doi:10.3389/fpubh.2025.1600196)
Supplement: Supplementary file 1 [file Table_1.docx]

**Table S1.Association between urine nicotine levels and physical health among boys**

| Physical health | Nicotine(ng/mL) | | | | | | |
| --- | --- | --- | --- | --- | --- | --- | --- |
|  | Total | | T1(≤3.33) | T2(3.33～6.27) | | T3(＞6.10) | |
|  | *β*(95%*CI*) | *P -*Value |  | *β*(95%*CI*) | *P -*Value | *β*(95%*CI*) | *P -*Value |
| Height(cm) |  |  |  |  |  |  |  |
| model1 | **0.392(0.202,0.581)** | **<0.001** | 1 | 1.382(-0.912,3.675) | 0.238 | **3.444(1.202,5.687)** | **0.003** |
| model2 | 0.170(-0.021,0.361) | 0.081 | 1 | -0.198(-2.383,1.988) | 0.859 | 1.460(-0.737,3.657) | 0.193 |
| Weight(kg) |  |  |  |  |  |  |  |
| model1 | **0.448(0.1750.721,)** | **0.001** | 1 | 2.658(-0.635,5.950) | 0.114 | **3.597(0.378,6.817)** | **0.029** |
| model2 | 0.220(-0.067,0.507) | 0.132 | 1 | 1.23(-2.067,4.528) | 0.465 | 1.594(-1.721,4.909) | 0.346 |
| BMI(kg/m2) |  |  |  |  |  |  |  |
| model1 | **0.120(0.020,0.221)** | **0.019** | 1 | 0.970(-0.242,2.181) | 0.117 | 0.930(-0.254,2.115) | 0.124 |
| model2 | 0.069(-0.038,0.176) | 0.205 | 1 | 0.715(-0.519,1.949) | 0.256 | 0.499(-0.742,1.739) | 0.431 |
| SBP(mmHg) |  |  |  |  |  |  |  |
| model1 | 0.074(-0.235,0.384) | 0.637 | 1 | 3.107(-0.634,6.848) | 0.104 | 2.095(-1.562,5.753) | 0.261 |
| model2 | 0.017(-0.319,0.353) | 0.921 | 1 | 2.800(-1.053,6.654) | 0.154 | 1.773(-2.101,5.647) | 0.370 |
| DBP(mmHg) |  |  |  |  |  |  |  |
| model1 | 0.032(-0.197,0.261) | 0.785 | 1 | 1.552(-1.232,4.336) | 0.275 | 1.067(-1.655,3.789) | 0.442 |
| model2 | 0.015(-0.234,0.264) | 0.908 | 1 | 1.643(-1.225,4.511) | 0.262 | 0.863(-2.020,3.746) | 0.557 |
| Waist circumference(cm) |  |  |  |  |  |  |  |
| model1 | **0.483(0.220,0.746)** | **<0.001** | 1 | **3.257(0.046,6.467)** | **0.047** | **3.890(0.751,7.029)** | **0.015** |
| model2 | 0.384(0.100,0.667) | 0.008 | 1 | 2.621(-0.661,5.903) | 0.118 | 2.930(-0.370,6.229) | 0.082 |
| Vital capacity |  |  |  |  |  |  |  |
| model1 | 13.844(-5.08832.775,) | 0.152 | 1 | 118.971(-108.956,346.898) | 0.306 | **261.635(38.788,484.482)** | **0.021** |
| model2 | -1.955(-21.592,17.681) | 0.845 | 1 | -15.005(-240.003,209.992) | 0.896 | 130.378(-95.789,356.546) | 0.259 |
| Vision left |  |  |  |  |  |  |  |
| model1 | 0.006(-0.004,0.015) | 0.253 | 1 | 0.070(-0.044,0.184) | 0.232 | -0.005(-0.116,0.107) | 0.933 |
| model2 | 0.002(-0.009,0.012) | 0.755 | 1 | 0.044(-0.074,0.161) | 0.464 | -0.039(-0.157,0.079) | 0.518 |
| Vision right |  |  |  |  |  |  |  |
| model1 | 0.004(-0.006,0.013) | 0.466 | 1 | 0.044(-0.069,0.157) | 0.477 | -0.043(-0.153,0.068) | 0.447 |
| model2 | 0.000(-0.010,0.011) | 0.938 | 1 | 0.022(-0.094,0.138) | 0.708 | -0.072(-0.188,0.045) | 0.227 |

Model 1: unadjusted.

Model 2 : adjusted for age, Caregiver's smoking status, fried food intake, meat intake, physical activity time , screen time.

**Table S2.Association between urine nicotine levels and physical health among girls**

| Physical health | Nicotine(ng/mL) | | | | | | |  |
| --- | --- | --- | --- | --- | --- | --- | --- | --- |
|  | Total | | T1(≤3.01) | T2(3.01～5.95) | | T3(＞5.95) | |  |
|  | *β*(95%*CI*) | *P -*Value |  | *β*(95%*CI*) | *P -*Value | *β*(95%*CI*) | *P -*Value |  |
| Height(cm) |  |  |  |  |  |  |  |  |
| model1 | **0.591(0.350,0.832)** | **<0.001** | 1 | **3.218(0.650,5.786)** | **0.014** | **6.022(3.427,8.617)** | **<0.001** |  |
| model2 | 0.096(-0.134,0.326) | 0.413 | 1 | 0.421(-1.822,2.665) | 0.713 | 1.251(-1.194,3.696) | 0.316 |  |
| Weight(kg) |  |  |  |  |  |  |  |  |
| model1 | **0.834(0.564,1.105)** | **<0.001** | 1 | 2.105(-0.7424.953,) | 0.147 | **8.065(5.188,10.942)** | **<0.001** |  |
| model2 | 0.473(0.186,0.761) | 0.001 | 1 | -0.016(-2.795,2.764) | 0.991 | 4.420(1.391,7.450) | 0.004 |  |
| BMI(kg/m2) |  |  |  |  |  |  |  |  |
| model1 | **0.239(0.138,0.339)** | **<0.001** | 1 | 0.260(-0.794,1.314) | 0.629 | **2.304(1.239,3.369)** | **<0.001** |  |
| model2 | 0.193(0.080,0.306) | ＜0.001 | 1 | -0.023(-1.114,1.069) | 0.967 | 1.813(0.624,3.003) | 0.003 |  |
| SBP(mmHg) |  |  |  |  |  |  |  |  |
| model1 | **0.736(0.369,1.104))** | **＜0.001** | 1 | 2.811(-1.062,6.683) | 0.155 | **7.377(3.464,11.291)** | **<0.001** |  |
| model2 | 0.590(0.177,1.003) | 0.005 | 1 | 1.626(-2.388,5.639) | 0.427 | 5.704(1.330,10.079) | 0.011 |  |
| DBP(mmHg) |  |  |  |  |  |  |  |  |
| model1 | **0.341(0.082,0.601)** | **0.010** | 1 | 0.799(-1.919,3.517) | 0.564 | **3.826(1.080,6.573)** | **0.006** |  |
| model2 | 0.237(-0.054,0.527) | 0.110 | 1 | 0.006(-2.789,2.802) | 0.997 | 2.267(-0.779,5.314) | 0.145 |  |
| Waist circumference(cm) |  |  |  |  |  |  |  |  |
| model1 | **0.309(0.057,0.561)** | **0.016** | 1 | -0.327(-2.946,2.291) | 0.806 | **2.965(0.320,5.611)** | **0.028** |  |
| model2 | 0.276(-0.008,0.561) | 0.057 | 1 | -0.595(-3.336,2.147) | 0.671 | 2.401(-0.587,5.389) | 0.115 |  |
| Vital capacity |  |  |  |  |  |  |  |  |
| model1 | **32.833(16.273,49.393)** | **<0.001** | 1 | 95.260(-82.404,272.924) | 0.293 | **317.864(138.332,497.397)** | **<0.001** |  |
| model2 | 15.428(-2.070,32.926) | 0.084 | 1 | -22.208(-193.307,148.891) | 0.799 | 122.972(-63.512,309.456) | 0.196 |  |
| Vision left |  |  |  |  |  |  |  |  |
| model1 | 0.007(-0.005,0.019) | 0.276 | 1 | -0.020(-0.147,0.108) | 0.763 | 0.066(-0.062,0.195) | 0.313 |  |
| model2 | 0.006(-0.007,0.020) | 0.352 | 1 | -0.012(-0.141,0.117) | 0.855 | 0.066(-0.075,0.207) | 0.358 |  |
| Vision right |  |  |  |  |  |  |  |  |
| model1 | -0.004(-0.016,0.009) | 0.548 | 1 | -0.067(-0.196,0.062) | 0.306 | -0.010(-0.140,0.121) | 0.884 |  |
| model2 | -0.009(-0.023,0.005) | 0.191 | 1 | -0.080(-0.213,0.052) | 0.236 | -0.060(-0.205,0.084) | 0.413 |  |

**Table S3.Association between urine cotinine levels and physical health among boys**

| Physical health | Cotinine(ng/mL) | | | | | | |
| --- | --- | --- | --- | --- | --- | --- | --- |
|  | Total | | T1(≤2.65) | T2(2.65～6.03) | | T3(＞6.03) | |
|  | *β*(95%*CI*) | *P -*Value |  | *β*(95%*CI*) | *P -*Value | *β*(95%*CI*) | *P -*Value |
| Height(cm) |  |  |  |  |  |  |  |
| model1 | -0.438(-0.892,0.016) | 0.058 | 1 | 0.684(-1.868,3.237) | 0.599 | **-3.898(-7.089,-0.707)** | **0.017** |
| model2 | 0.176(-0.608,0.255) | 0.424 | 1 | 1.130(-1.244,3.503) | 0.351 | -1.532(-4.562,1.499) | 0.322 |
| Weight(kg) |  |  |  |  |  |  |  |
| model1 | **-0.681(-1.334,-0.028)** | **0.041** | 1 | 2.216(-1.449,5.881) | 0.236 | -4.043(-8.624,0.537) | 0.084 |
| model2 | -0.539(-1.188,0.110) | 0.103 | 1 | 2.102(-1.479,5.684) | 0.250 | -2.399(-6.972,2.174) | 0.304 |
| BMI(kg/m2) |  |  |  |  |  |  |  |
| model1 | -0.231(-0.470,0.009) | 0.059 | 1 | 0.712(-0.636,2.060) | 0.301 | -1.099(-2.784,0.586) | 0.201 |
| model2 | -0.228(-0.470,0.015) | 0.065 | 1 | 0.554(-0.786,1.895) | 0.418 | -0.919(-2.630,0.793) | 0.293 |
| SBP(mmHg) |  |  |  |  |  |  |  |
| model1 | 0.027(-0.713,0.767) | 0.943 | 1 | -1.265(-5.429,2.899) | 0.551 | 0.164(-5.040,5.369) | 0.951 |
| model2 | 0.150(-0.611,0.910) | 0.700 | 1 | -1.045(-5.231,3.141) | 0.625 | 1.289(-4.056,6.633) | 0.637 |
| DBP(mmHg) |  |  |  |  |  |  |  |
| model1 | 0.168(-0.381,0.716) | 0.548 | 1 | -0.576(-3.674,2.523) | 0.716 | 0.763(-3.110,4.636) | 0.700 |
| model2 | 0.210(-0.353,0.773) | 0.465 | 1 | -0.437(-3.552,2.678) | 0.783 | 1.167(-2.810,5.144) | 0.565 |
| Waist circumference(cm) |  |  |  |  |  |  |  |
| model1 | -0.261(-0.890,0.369) | 0.417 | 1 | 2.237(-1.337,5.810) | 0.220 | -0.305(-4.771,4.162) | 0.894 |
| model2 | -0.219(-0.861,0.422) | 0.503 | 1 | 2.129(-1.436,5.694) | 0.242 | 0.600(-3.952,5.152) | 0.796 |
| Vital capacity |  |  |  |  |  |  |  |
| model1 | -24.794(-70.067,20.480) | 0.283 | 1 | -55.723(-309.437,197.991) | 0.667 | -234.494(-551.600,82.612) | 0.147 |
| model2 | -11.212(-55.621,33.197) | 0.621 | 1 | -45.487(-289.874,198.900) | 0.715 | -101.187(-413.228,210.854) | 0.525 |
| Vision left |  |  |  |  |  |  |  |
| model1 | -0.008(-0.031,0.015) | 0.472 | 1 | 0.092(-0.035,0.219) | 0.154 | 0.017(-0.142,0.175) | 0.836 |
| model2 | -0.006(-0.029,0.018) | 0.636 | 1 | 0.086(-0.0420.213,) | 0.187 | 0.034(-0.129,0.196) | 0.686 |
| Vision right |  |  |  |  |  |  |  |
| model1 | -0.013(-0.036,0.009) | 0.243 | 1 | 0.055(-0.071,0.181) | 0.390 | -0.018(-0.175,0.139) | 0.824 |
| model2 | -0.013(-0.036,0.010) | 0.269 | 1 | 0.041(-0.084,0.167) | 0.519 | -0.012(-0.172,0.149) | 0.885 |

Model 1: unadjusted.

Model 2 : adjusted for age, Caregiver's smoking status, fried food intake, meat intake, physical activity time , screen time.

**Table S4.Association between urine cotinine levels and physical health among girls**

| Physical health | Cotinine(ng/mL) | | | | | | |
| --- | --- | --- | --- | --- | --- | --- | --- |
|  | Total | | T1(≤2.35) | T2(2.35～5.32) | | T3(＞5.32) | |
|  | *β*(95%*CI*) | *P -*Value |  | *β*(95%*CI*) | *P -*Value | *β*(95%*CI*) | *P -*Value |
| Height(cm) |  |  |  |  |  |  |  |
| model1 | -1.145(-1.754,-0.536) | <0.001 | 1 | -2.608(-5.373,0.157) | 0.064 | -4.561(-8.488,-0.634) | 0.023 |
| model2 | -0.695(-1.239,-0.150) | 0.012 | 1 | -2.364(-4.671,-0.058) | 0.045 | -3.669(-7.010,-0.328) | 0.031 |
| Weight(kg) |  |  |  |  |  |  |  |
| model1 | -0.216(-0.898,0.467) | 0.536 | 1 | -1.923(-4.988,1.143) | 0.219 | 0.090(-4.264,4.444) | 0.968 |
| model2 | 0.073(-0.606,0.753) | 0.833 | 1 | -1.734(-4.592,1.124) | 0.234 | 0.848(-3.292,4.988) | 0.688 |
| BMI(kg/m2) |  |  |  |  |  |  |  |
| model1 | 0.162(-0.0920.416,) | 0.212 | 1 | -0.288(-1.423,0.847) | 0.619 | 1.000(-0.612,2.611) | 0.224 |
| model2 | 0.182(-0.085,0.449) | 0.181 | 1 | -0.277(-1.400,0.845) | 0.628 | 1.130(-0.496,2.755) | 0.173 |
| SBP(mmHg) |  |  |  |  |  |  |  |
| model1 | 0.096(-0.832,1.023) | 0.840 | 1 | 0.463(-3.707,4.633) | 0.828 | -0.063(-5.985,5.858) | 0.983 |
| model2 | 0.248(-0.729,1.225) | 0.619 | 1 | 0.583(-3.544,4.710) | 0.782 | 0.545(-5.432,6.523) | 0.858 |
| DBP(mmHg) |  |  |  |  |  |  |  |
| model1 | -0.048(-0.703,0.607) | 0.885 | 1 | -0.597(-3.523,2.330) | 0.689 | -1.258(-5.414,2.898) | 0.553 |
| model2 | -0.005(-0.691,0.681) | 0.989 | 1 | -0.591(-3.466,2.283) | 0.687 | -0.825(-4.988,3.339) | 0.698 |
| Waist circumference(cm) |  |  |  |  |  |  |  |
| model1 | 0.388(-0.247,1.023) | 0.231 | 1 | -1.061(-3.880,1.758) | 0.461 | 2.877(-1.126,6.880) | 0.159 |
| model2 | 0.369(-0.304,1.043) | 0.282 | 1 | -0.917(-3.736,1.902) | 0.524 | 2.929(-1.153,7.012) | 0.160 |
| Vital capacity |  |  |  |  |  |  |  |
| model1 | -51.015(-92.808,-9.223) | 0.017 | 1 | -64.222(-255.526,127.082) | 0.511 | -137.339(-409.011,134.333) | 0.322 |
| model2 | -34.023(-75.416,7.370) | 0.107 | 1 | -49.399(-225.331,126.534) | 0.582 | -83.475(-338.295,171.344) | 0.521 |
| Vision left |  |  |  |  |  |  |  |
| model1 | -0.012(-0.042,0.019) | 0.446 | 1 | -0.051(-0.188,0.086) | 0.468 | -0.097(-0.292,0.098) | 0.328 |
| model2 | -0.008(-0.040,0.024) | 0.619 | 1 | -0.056(-0.189,0.077) | 0.413 | -0.079(-0.272,0.114) | 0.422 |
| Vision right |  |  |  |  |  |  |  |
| model1 | -0.001(-0.032,0.030) | 0.942 | 1 | -0.024(-0.1630.115,) | 0.738 | -0.013(-0.210,0.184) | 0.897 |
| model2 | 0.006(-0.026,0.038) | 0.706 | 1 | -0.020(-0.156,0.117) | 0.777 | 0.011(-0.186,0.208) | 0.912 |

Model 1: unadjusted.

Model 2 : adjusted for age, Caregiver's smoking status, fried food intake, meat intake, physical activity time , screen time.

**Table S5.Association between urine trans-3'-hydroxycotinine levels and physical health among boys**

| Physical health | Trans-3'-hydroxycotinine(ng/mL) | | | | | | |
| --- | --- | --- | --- | --- | --- | --- | --- |
|  | Total | | T1(≤3.19) | T2(3.19～12.45) | | T3(＞12.45) | |
|  | *β*(95%*CI*) | *P -*Value |  | *β*(95%*CI*) | *P -*Value | *β*(95%*CI*) | *P -*Value |
| Height(cm) |  |  |  |  |  |  |  |
| model1 | -0.152(-0.358,0.054) | 0.148 | 1 | -1.872(-4.410,0.667) | 0.148 | -2.272(-5.443,0.899) | 0.160 |
| model2 | -0.054(-0.249,0.140) | 0.584 | 1 | -1.475(-3.830,0.880) | 0.220 | -0.061(-3.073,2.950) | 0.968 |
| Weight(kg) |  |  |  |  |  |  |  |
| model1 | -0.024(-0.320,0.273) | 0.876 | 1 | -2.026(-5.671,1.619) | 0.276 | -2.015(-6.568,2.538) | 0.386 |
| model2 | 0.062(-0.231,0.355) | 0.677 | 1 | -1.572(-5.126,1.982) | 0.386 | -0.164(-4.709,4.380) | 0.943 |
| BMI(kg/m2) |  |  |  |  |  |  |  |
| model1 | 0.033(-0.076,0.142) | 0.551 | 1 | -0.362(-1.703,0.979) | 0.597 | -0.337(-2.012,1.337) | 0.693 |
| model2 | 0.050(-0.060,0.159) | 0.374 | 1 | -0.247(-1.577,1.083) | 0.716 | -0.021(-1.721,1.679) | 0.981 |
| SBP(mmHg) |  |  |  |  |  |  |  |
| model1 | -0.043(-0.379,0.293) | 0.804 | 1 | -1.359(-5.500,2.782) | 0.520 | -1.356(-6.529,3.816) | 0.607 |
| model2 | 0.023(-0.320,0.367) | 0.894 | 1 | -1.002(-5.155,3.152) | 0.636 | -0.099(-5.410,5.211) | 0.971 |
| DBP(mmHg) |  |  |  |  |  |  |  |
| model1 | -0.106(-0.355,0.143) | 0.403 | 1 | 0.264(-2.817,3.346) | 0.866 | -0.822(-4.672,3.027) | 0.675 |
| model2 | -0.127(-0.382,0.127) | 0.326 | 1 | 0.180(-2.911,3.271) | 0.909 | -0.900(-4.852,3.052) | 0.655 |
| Waist circumference(cm) |  |  |  |  |  |  |  |
| model1 | -0.029(-0.315,0.257) | 0.841 | 1 | -1.972(-5.525,1.582) | 0.277 | -2.770(-7.209,1.669) | 0.221 |
| model2 | 0.038(-0.251,0.328) | 0.795 | 1 | -1.574(-5.112,1.963) | 0.383 | -1.424(-5.947,3.099) | 0.537 |
| Vital capacity |  |  |  |  |  |  |  |
| model1 | -1.887(-22.444,18.671) | 0.857 | 1 | -120.546(-372.852,131.760) | 0.349 | -44.310(-359.467,270.848) | 0.783 |
| model2 | 7.259(-12.793,27.310) | 0.478 | 1 | -75.584(-318.067,166.899) | 0.541 | 136.263(-173.782,446.308) | 0.389 |
| Vision left |  |  |  |  |  |  |  |
| model1 | 0.002(-0.008,0.013) | 0.677 | 1 | 0.103(-0.023,0.229) | 0.111 | -0.035(-0.193,0.123) | 0.663 |
| model2 | 0.003(-0.007,0.014) | 0.560 | 1 | 0.112(-0.015,0.238) | 0.083 | -0.012(-0.173,0.150) | 0.888 |
| Vision right |  |  |  |  |  |  |  |
| model1 | 0.005(-0.005,0.015) | 0.334 | 1 | **0.131(0.006,0.256)** | **0.040** | 0.023(-0.133,0.179) | 0.775 |
| model2 | 0.006(-0.004,0.017) | 0.229 | 1 | 0.145(0.020,0.269) | 0.023 | 0.050(-0.110,0.209) | 0.543 |

Model 1: unadjusted.

Model 2 : adjusted for age, Caregiver's smoking status, fried food intake, meat intake, physical activity time , screen time.

**Table S6.Association between urine trans-3'-hydroxycotinine levels and physical health among girls**

| Physical health | Trans-3'-hydroxycotinine(ng/mL) | | | | | | |
| --- | --- | --- | --- | --- | --- | --- | --- |
|  | Total |  | T1(≤2.78) | T2(2.78～11.27) | | T3(＞11.27) | |
|  | *β*(95%*CI*) | *P -*Value |  | *β*(95%*CI*) | *P -*Value | *β*(95%*CI*) | *P -*Value |
| Height(cm) |  |  |  |  |  |  |  |
| model1 | 0.085(-0.185,0.354) | 0.538 | 1 | 0.562(-2.126,0.168) | 0.682 | -2.984(-6.911,0.942) | 0.136 |
| model2 | 0.180(-0.049,0.410) | 0.124 | 1 | 0.913(-1.365,3.192) | 0.432 | 1.450(-1.941,4.840) | 0.402 |
| Weight(kg) |  |  |  |  |  |  |  |
| model1 | -0.121(-0.422,0.181) | 0.432 | 1 | 1.364(-1.616,4.344) | 0.370 | -4.135(-8.489,0.218) | 0.063 |
| model2 | -0.046(-0.332,0.241) | 0.754 | 1 | 1.529(-1.293,4.352) | 0.288 | -1.162(-5.362,3.039) | 0.588 |
| BMI(kg/m2) |  |  |  |  |  |  |  |
| model1 | -0.076(-0.189,0.036) | 0.182 | 1 | 0.434(-0.669,1.537) | 0.440 | -1.143(-2.755,0.468) | 0.164 |
| model2 | -0.063(-0.175,0.050) | 0.273 | 1 | 0.430(-0.678,1.538) | 0.447 | -0.858(-2.508,0.791) | 0.308 |
| SBP(mmHg) |  |  |  |  |  |  |  |
| model1 | 0.010(-0.400,0.420) | 0.962 | 1 | -2.276(-6.329,1.777) | 0.271 | -0.763(-6.684,5.159) | 0.801 |
| model2 | 0.040(-0.372,0.452) | 0.848 | 1 | -2.210(-6.286,1.866) | 0.288 | 0.476(-5.590,6.541) | 0.878 |
| DBP(mmHg) |  |  |  |  |  |  |  |
| model1 | 0.279(-0.011,0.568) | 0.059 | 1 | -0.372(-3.217,2.472) | 0.798 | **4.251(0.095,8.407)** | **0.045** |
| model2 | 0.323(0.034,0.613) | 0.028 | 1 | -0.665(-3.503,2.174) | 0.646 | 5.253(1.029,9.478) | 0.015 |
| Waist circumference(cm) |  |  |  |  |  |  |  |
| model1 | -0.105(-0.386,0.176) | 0.463 | 1 | 1.699(-1.041,4.439) | 0.224 | -1.659(-5.662,2.345) | 0.417 |
| model2 | -0.111(-0.395,0.172) | 0.442 | 1 | 1.560(-1.224,4.344) | 0.272 | -1.626(-5.769,2.517) | 0.442 |
| Vital capacity |  |  |  |  |  |  |  |
| model1 | 5.413(-13.058,23.885) | 0.566 | 1 | -29.667(-215.610,156.277) | 0.755 | -120.344(-392.004,151.315) | 0.385 |
| model2 | 8.279(-9.172,25.730) | 0.352 | 1 | -54.239(-227.989,119.510) | 0.541 | 35.037(-223.535,293.610) | 0.791 |
| Vision left |  |  |  |  |  |  |  |
| model1 | 0.006(-0.008,0.019) | 0.398 | 1 | 0.016(-0.118,0.149) | 0.819 | 0.104(-0.091,0.299) | 0.295 |
| model2 | 0.007(-0.006,0.020) | 0.305 | 1 | 0.058(-0.074,0.189) | 0.390 | 0.149(-0.047,0.344) | 0.136 |
| Vision right |  |  |  |  |  |  |  |
| model1 | 0.008(-0.006,0.022) | 0.260 | 1 | -0.6263E-5(-0.135,0.135) | 0.999 | 0.109(-0.088,0.306) | 0.278 |
| model2 | 0.009(-0.004,0.023) | 0.189 | 1 | 0.027(-0.107,0.162) | 0.693 | 0.167(-0.033,0.367) | 0.102 |

Model 1: unadjusted.

Model 2 : adjusted for age, Caregiver's smoking status, fried food intake, meat intake, physical activity time , screen time.
